# *Abcc5* Knockout Mice Have Lower Fat Mass and Increased Levels of Circulating GLP‐1

**DOI:** 10.1002/oby.22521

**Published:** 2019-07-24

**Authors:** Malgorzata Cyranka, Anna Veprik, Eleanor J. McKay, Nienke van Loon, Amber Thijsse, Luke Cotter, Nisha Hare, Affan Saibudeen, Swathi Lingam, Elisabete Pires, Pierre Larraufie, Frank Reimann, Fiona Gribble, Michelle Stewart, Elizabeth Bentley, Pamela Lear, James McCullagh, James Cantley, Roger D. Cox, Heidi de Wet

**Affiliations:** ^1^ Department of Physiology, Anatomy and Genetics University of Oxford Oxford UK; ^2^ Chemistry Research Laboratory University of Oxford Oxford UK; ^3^ Wellcome Trust‐MRC Institute of Metabolic Science Addenbrooke's Hospital Cambridge UK; ^4^ MRC Harwell Institute, Genetics of Type 2 Diabetes Mammalian Genetics Unit, Harwell Campus Oxfordshire UK

## Abstract

**Objective:**

A previous genome‐wide association study linked overexpression of an ATP‐binding cassette transporter, *ABCC5*, in humans with a susceptibility to developing type 2 diabetes with age. Specifically, *ABCC5* gene overexpression was shown to be strongly associated with increased visceral fat mass and reduced peripheral insulin sensitivity. Currently, the role of ABCC5 in diabetes and obesity is unknown. This study reports the metabolic phenotyping of a global *Abcc5* knockout mouse.

**Methods:**

A global *Abcc5^‐/‐^* mouse was generated by CRISPR/Cas9. Fat mass was determined by weekly EchoMRI and fat pads were dissected and weighed at week 18. Glucose homeostasis was ascertained by an oral glucose tolerance test, intraperitoneal glucose tolerance test, and intraperitoneal insulin tolerance test. Energy expenditure and locomotor activity were measured using PhenoMaster cages. Glucagon‐like peptide 1 (GLP‐1) levels in plasma, primary gut cell cultures, and GLUTag cells were determined by enzyme‐linked immunosorbent assay.

**Results:**

*Abcc5^‐/‐^* mice had decreased fat mass and increased plasma levels of GLP‐1, and they were more insulin sensitive and more active. Recombinant overexpression of ABCC5 protein in GLUTag cells decreased GLP‐1 release.

**Conclusions:**

ABCC5 protein expression levels are inversely related to fat mass and appear to play a role in the regulation of GLP‐1 secretion from enteroendocrine cells.

## Introduction

Hormones secreted by enteroendocrine cells, the endocrine cells of the gut, are central to the regulation of gastrointestinal physiology, energy metabolism, and appetite 1. In particular, the central role of gut hormone action on energy metabolism has been demonstrated by bariatric surgery outcomes, which show that increased gut hormone release contributes to the reversal of pathological insulin resistance in those with type 2 diabetes (T2D) within days following Roux‐en‐Y bypass surgery 2, 3, 4. The incretin hormone glucagon‐like peptide 1 (GLP‐1), which potentiates insulin release from pancreatic β‐cells, plays a pivotal role in the changes observed in glucose handling post surgery 5, 6. GLP‐1 has multiple actions on peripheral tissues, such as driving cell proliferation in the pancreas and being both antiapoptotic and neuroprotective 7. GLP‐1 receptor (GLP‐1R) activation leads to enhanced satiety, weight loss, decreased glucose production in the liver, and enhanced insulin sensitivity in skeletal muscle; GLP‐1R agonists are currently in clinical use for the treatment of T2D 7.

A genome‐wide association study (GWAS) linked elevated expression of an ATP‐binding cassette transporter, *ABCC5*, in subcutaneous adipose tissue to reduced peripheral insulin sensitivity in nondiabetic individuals with associated increased visceral fat accumulation and a threefold increased risk of developing T2D with age 8. This trend was observed in populations of disparate ancestry. The role of ABCC5 in diabetes and obesity remains unexplored, and any link between ABCC5 overexpression and increased fat stores is unknown.

The ABC transporters are a large family of membrane ATPases best known for their roles in multidrug resistance observed in chemotherapy‐resistant tumors 9. However, these transporters fulfill many other essential functions, such as antigen presentation to the immune system (TAP1/ABCB2) 10, 11, Cl^‐ ^ion permeability of the cell membrane (CFTR/ABCC7) 12, and the regulation of insulin release by adenosine nucleotides and sulfonylurea drugs from pancreatic β‐cells by the sulfonylurea receptor (SUR1/ABCC8), which forms the K_ATP_ channel complex along with inward rectifier K(+) channel Kir6.2 13, 14, 15. The role of ABCC5 transporter activity in mammals is currently unknown, but knocking *Abcc5* gene expression out in animal models indicated a role for this protein in heme transport in *Caenorhabditis elegans* and hind gut formation in sea urchins 16, 17. Jansen et al. 18 demonstrated that ABCC5 is a glutamate conjugate transporter, and tissues of the knockout mouse were shown to accumulate up to eight different glutamate metabolites, including the inhibitory neuropeptides N‐acetylaspartylglutamate (NAAG) and N‐acetylaspartyldiglutamate (NAAG_2_).

This study reports the metabolic phenotyping of *Abcc5* knockout mice, *Abcc5^‐/‐^*. Our work demonstrated that ABCC5 protein expression plays a central role in energy metabolism in mammals, with *Abcc5^‐/‐^* mice showing lower white and brown adipose tissue and increased GLP‐1 release from enteroendocrine cells of the small intestine.

## Methods

### CRISPR/Cas9 *Abcc5* knockout mice

The *Abcc5^‐/‐^* CRISPR/Cas9 mice were generated on a B6N background and were obtained from the Medical Research Council (MRC) Harwell Institute, which distributes these mice on behalf of the European Mouse Mutant Archive (www.infrafrontier.eu). The protospacer sequences used to knock out the *Abcc5* gene through direct injection into blastocysts were *Abcc5*_5’, 5’‐GCTGTGGGTTGCTGATTGCA GGG‐3’; and *Abcc5*_3’, 5’‐CTTCTCTCACACATAGCCAAAGG‐3’.

### Metabolic phenotyping

All animal studies were approved by the MRC Harwell Institute Ethical Review Committee, and all procedures were carried out within license restrictions (PPL 30/3146) under the Animal (Scientific Procedures) Act 1986, issued by the UK Government Home Office Department. *Abcc5^‐/‐^* mice and wild‐type littermate controls were kept in accordance with Home Office welfare guidance (12 hours of light and dark cycles; temperature 21°C ± 2°C and humidity 55% ± 10% at the Mary Lyon Centre animal facility, MRC Harwell, Oxfordshire, UK). Mice had free access to water (10 parts per million chlorine) and were fed ad libitum on standard chow (RM3; Special Diet Services, Essex, UK). All *in vivo* studies were performed on mice aged 4 to 18 weeks.

### Body mass and composition

Body mass was measured for two independent cohorts of mice at baseline (week 4) and weekly thereafter on scales calibrated to 0.01 g. Whole body composition (fat and lean mass) was determined using an EchoMRI‐136 Body Composition Analyzer for Live Small Animals (Echo Medical Systems, Houston, Texas) at baseline (week 4) and weekly thereafter for *Abcc5^‐/‐^* mice and wild‐type littermate controls.

### Glucose tolerance tests

#### Oral glucose tolerance test

Twelve‐week‐old *Abcc5^‐/‐^* mice and wild‐type littermate controls were fasted overnight (16 hours). The mice were weighed and fasting glucose levels measured from whole blood via tail bleed under local anesthesia (5% EMLA cream (lidocaine/prilocaine), AstraZeneca, Cambridge, UK). An oral gavage of 20% glucose solution in 0.9% NaCl at 2 g/kg of body mass was administered and whole blood glucose measurements taken at 15 minutes, 30 minutes, 60 minutes, and 120 minutes after the gavage. The glucose measurements were performed using a handheld AlphaTRAK glucometer for pets (Abbott Laboratories, Lake Bluff, Illinois).

#### Intraperitoneal glucose tolerance test

Thirteen‐week‐old *Abcc5^‐/‐^* mice and wild‐type littermate controls were processed as for the oral glucose tolerance test. Intraperitoneal injections of 20% glucose solution in 0.9% NaCl at 2 g/kg of body mass were administered and whole blood glucose samples taken at 15 minutes, 30 minutes, 60 minutes, and 120 minutes via tail bleed.

### Insulin tolerance test

At week 14, *Abcc5^‐/‐^* mice and wild‐type littermate controls were fasted for 4 hours during the light phase. The mice were weighed, and whole blood samples were collected at time 0 via tail vein for baseline glucose measurements. Intraperitoneal injections of 0.5 IU/kg per mouse (females) or 1.0 IU/kg per mouse (males) of insulin diluted in 0.9% NaCl in sterile water were made, and subsequent blood samples were taken at 15 minutes, 30 minutes, 45 minutes, 60 minutes, and 90 minutes. Blood glucose uptake measurements were taken using an AlphaTRAK glucometer.

### Indirect calorimetry (energy expenditure, locomotor activity)

At week 12, *Abcc5^‐/‐^* mice and wild‐type littermate controls were individually housed in PhenoMaster cages (TSE Systems, Bad Homburg, Germany) for collection of energy intake‐ and expenditure‐related data over 24 hours. The cage system included photobeam‐based activity monitoring that records ambulatory movements in the horizontal and vertical planes. An indirect gas calorimetry system simultaneously measured oxygen consumption (VO_2_), carbon dioxide production (VCO_2_), and respiratory exchange ratio (RER).

### Adipose tissue harvest and Western blots

Epididymal white adipose tissue (epiWAT), periovarian white adipose tissue (periWAT), and interscapular brown adipose tissue (iBAT) were collected as described previously 19 and immediately weighed. All tissues were snap frozen in liquid nitrogen and stored at −80°C. Tissue processing is detailed in the online Supporting Information.

### siRNA *Abcc5* gene knockdown and recombinant ABCC5 protein overexpression in GLUTag cells

GLUTag cells were used with permission of Professor Daniel Drucker (Toronto, Ontario, Canada). Cells were maintained in low‐glucose DMEM (11885084; Thermo Fisher Scientific, Waltham, Massachusetts) supplemented with 2mM glutamine (G7513‐100ML; MilliporeSigma, Burlington, Massachusetts), 100‐U/mL penicillin and 100‐µg/mL streptomycin (15140122; Thermo Fisher Scientific), and 10% fetal bovine serum (FBS, F7524‐500ML; MilliporeSigma) in tissue culture flasks coated with 0.4% Matrigel (354234, Corning Inc., Corning, New York). For knockdown and overexpression experiments, cells were seeded onto 24‐well plates coated with 0.4% Matrigel at the density of 0.05 × 10^6^ cells per well and incubated until 80% confluent. *Abcc5* small interfering RNA (siRNA) knockdown was performed with predesigned siRNA (AM16708, Ambion; Thermo Fisher Scientific) targeting reference *Abcc5* sequence (probe ID: 188572). *Abcc5* knockdown was achieved by Lipofectamine RNAiMAX Transfection Reagent (13778075; Thermo Fisher Scientific) according to the manufacturer’s protocol. Nontargeting siRNA (AM4611, Ambion; Thermo Fisher Scientific) was used as a negative control. ABCC5 protein overexpression was achieved by the branched polyethylenimine (408727‐100ML; MilliporeSigma)‐mediated transfection of the mammalian expression vector (pSF‐CMV‐Amp; Oxford Genetics, Oxford, UK) carrying a codon‐optimized human *ABCC5* gene sequence (custom synthesis; GenScript, Nanjing, China). Empty vector pSF‐CMV‐Amp was used as a negative control, and no effect on the expression of ABCC5 was observed. *ABCC5* gene expression was quantified by quantitative reverse transcription‐polymerase chain reaction (RT‐qPCR) (primers were *Abcc5*, forward CCTGCTGCGTCACTGTAAGA, reverse TCAAACTCCACCACCTGTCC; pSF‐CMV‐Amp‐*ABCC5*, forward CAGCGTATCTCTCTGGCTCG, reverse AGCACGGTCTTGGACTTCAG) followed by the protein analysis on Western blot (antibodies were goat polyclonal anti‐ABCC5, sc‐5781, 1:200; Santa Cruz Biotechnology, Dallas, Texas; and secondary goat anti‐rabbit IgG [H/L]:HRP, STAR124P, 1:10,000; Bio‐Rad Laboratories, Inc., Hercules, California). The complete Western blots are shown in Supporting Information Figure S1.

### GLP‐1 secretion assay

GLP‐1 secretion assay was similar for GLUTag cells and primary small intestine cultures. Cells were grown on Matrigel‐coated 24‐well plates until 80% confluent (GLUTag cells) or overnight (gut primary cultures). Cells were washed twice with warm phosphate‐buffered saline (PBS), treated with different glucose concentrations (0mM, 1mM, 6mM) prepared in Krebs buffer (138mM NaCl, 4.5mM KCl, 2.5mM CaCl_2_, 1.2mM MgCl_2_, 4.2mM NAHCO_3_, 1.2mM Na_2_HPO_4_/NaH_2_PO_4_, 10mM HEPES, supplemented with 0.1% fatty‐acid‐free bovine serum albumin (BSA, A6003‐10G; MilliporeSigma), and incubated for 2 hours at 37°C with 5% CO_2_. Supernatants were harvested into the Triton X‐100 and TWEEN 20 mixture (0.05% and 0.04% final concentrations, respectively) and spun down at 300*g* for 5 minutes to remove floating cells. The remaining cell monolayer was lysed in radioimmunoprecipitation assay buffer. Supernatants were analyzed for active GLP‐1 by enzyme‐linked immunosorbent assay (ELISA). Active GLP‐1 secretion from GLUTag cells was measured by two independent ELISA kits (EZGLPHS‐35K; MilliporeSigma; and 62GLPPEG; Cisbio, Codolet, France). Active GLP‐1 secretion from primary gut cultures was measured by fluorescence resonance energy transfer (FRET)‐based ELISA (62GLPPEG; Cisbio). All buffer reagents were from MilliporeSigma, and the glucose solution (A2494001) and HEPES buffer (15630056) were from Thermo Fisher Scientific.

### Isolation and culture of gut primary cells from *Abcc5^‐/‐^* mice

Mixed primary cultures of murine intestine isolated from *Abcc5^‐/‐^* mice and wild‐type littermate controls were prepared as previously described 20 and detailed in online Supporting Information.

### Blood chemistry analysis

Total cholesterol, HDL, LDL, glycerol, and triglyceride levels were determined in terminal lithium‐heparin plasma samples collected at 5 minutes, following an oral glucose gavage of overnight‐fasted *Abcc5^‐/‐^* mice and wild‐type littermate controls, using a Beckman Coulter AU680 clinical chemistry analyzer (Beckman Coulter, Inc., Brea, California) with reagents and settings recommended by the manufacturer.

### *Abcc5* gene expression analysis

ABC transporter expression profiles were determined by RNA sequencing as previously described 21. Briefly, fluorescent and nonfluorescent cells were isolated in triplicate by fluorescence‐activated cell sorting from the duodenum/jejunum (top 10 cm of small intestine), ileum (bottom 10 cm of small intestine), and colon of NeuroD1‐CrexRosa26EYFP or GLU‐Venus mice (Gribble/Reimann laboratory, Cambridge, UK), labeling all enteroendocrine cells or only proglucagon‐expressing cells, respectively. Total RNA (isolated with RNeasy Plus Micro Kit [Qiagen, Hilden, Germany] and amplified using Ovation RNA‐Seq System V2 [NuGEN Technologies, Inc., Redwood City, California]) was used to create barcoded libraries, which were sequenced using an Illumina HiSeq 2500 System (Illumina, Inc., San Diego, California) at the Genomics Core Facility of the Cancer Research UK Cambridge Institute. Sequence reads were demultiplexed using the Casava pipeline (Illumina) and then aligned to the mouse genome (GRCm38) using TopHat version 2.1.0 (Johns Hopkins University, Baltimore, Maryland). Gene expression (fragments per kilobase per million read FPKM) was determined using Cufflinks version 2.2.1 (http://cole-trapnell-lab.github.io/cufflinks/), and differential gene expression was assessed by DESeq2 (https://bioconductor.org/packages/release/bioc/html/DESeq2.html), excluding one GLU‐Venus duodenal fluorescently labeled data set because of apparent contamination.

### Metabolomics

Metabolites were extracted from approximately 5 × 10^6^ cells (grown in cell culture dishes) by the addition of 500 µL of ice‐cold 80% aqueous methanol. Supernatants were combined and filtered using a 3‐kDa ultrafilter (Millipore), dried in a SpeedVac (Thermo Fisher Scientific), and subsequently stored at −80°C. On the day of analysis, the dried extracts were reconstituted in 60 μL of ice‐cold 80% aqueous methanol. A quality control sample was made by combining 5 µL of each sample. Sample analysis was performed using anion‐exchange chromatography (Thermo UltiMate 3000 UHPLC, Thermo Fisher Scientific) coupled directly to a high‐resolution Orbitrap mass spectrometer (Q Exactive HF Hybrid Quadrupole‐Orbitrap, Thermo Fisher Scientific) as previously described 22 and detailed in online Supporting Information.

### Statistics

Data are presented as mean ± SEM. Simple pairwise comparisons were made using unpaired two‐tailed *t* tests. For sample numbers ≥ 10 to 15, a Student *t* test was used. For sample numbers < 10 or where unequal numbers of two groups were compared, the more stringent Welch’s unequal variances *t* test was used. Multiple comparisons were made using one‐ or two‐way ANOVA with a Bonferroni post hoc test. *P* < 0.05 for a 95% CI was regarded as statistically significant. Statistics were performed using GraphPad Prism 6 (Graphpad Software, La Jolla, California).

## Results

### *Abcc5^‐/‐^* mice have lower body mass because of decreased adiposity

**Figure 1:**
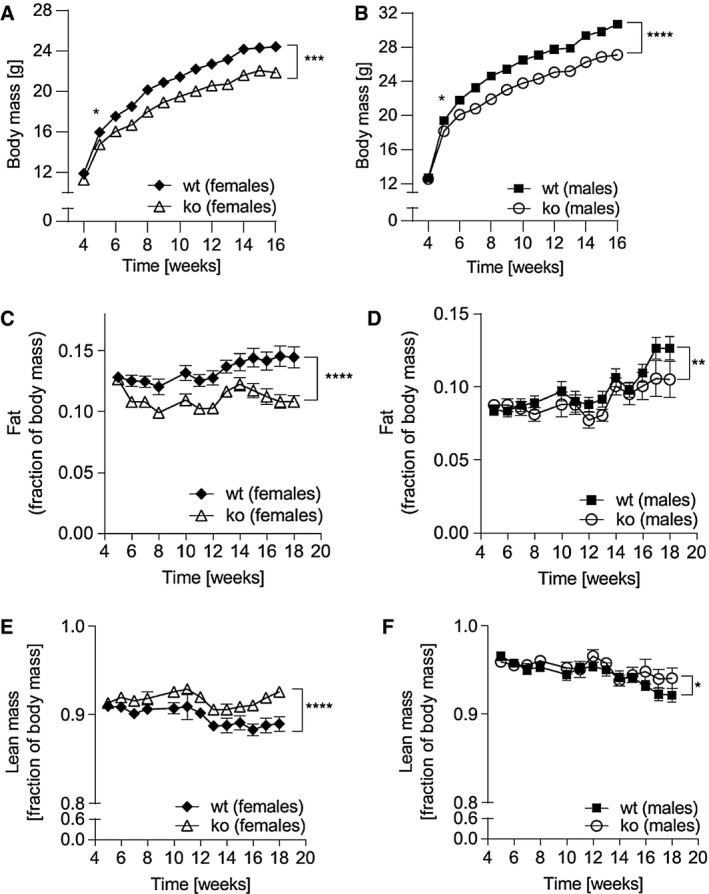
Fat mass and body composition of *Abcc5^‐/‐^* mice and wild‐type (wt) littermate controls. (**A**) Female and (**B**) male mice were weighed weekly from weaning over a period of 16 weeks. Data shown for two separate cohorts for male:female:wt:*Abcc5^‐/‐^* = 30:30:30:29. (**C,D**) Fat mass and (**E,F**) lean mass were recorded by EchoMRI for male:female:wt:*Abcc5^‐/‐^* = 15:15:15:15 from 4 weeks to 18 weeks. Each data point represents mean ± SEM. Changes in body mass, fat, and lean mass over time for *Abcc5^‐/‐^* vs. wt were analyzed by two‐way ANOVA with a Bonferroni post hoc test. *P < 0.05; **P ≤ 0.01; ***P ≤ 0.001; ****P ≤ 0.0001. Differences in body mass between *Abcc5^‐/‐^* and wt mice (both female and male) were statistically significant from week 5 and for all weeks onward when analyzed by a Welch’s unequal variances *t* test for that week; *P < 0.05. Data for panels C‐F are presented as a fraction of whole body mass measured at the same time point. Error bars smaller than the symbols are not visible on the graph.

**Figure 2:**
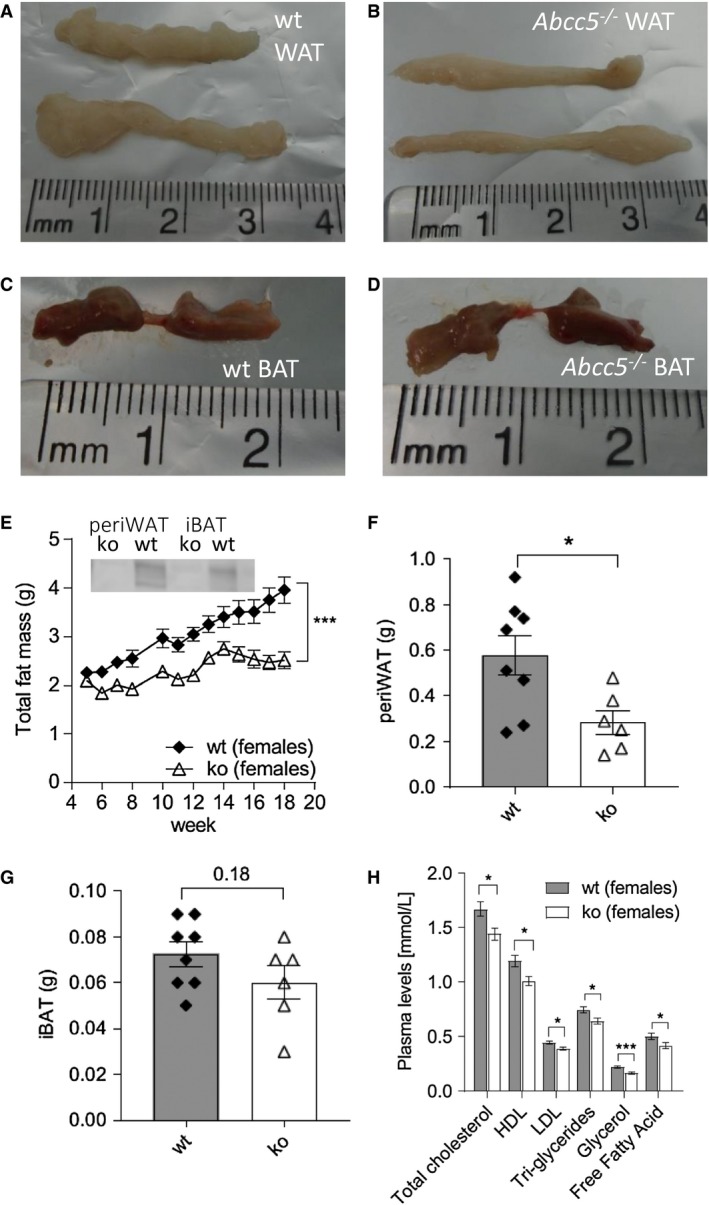
Female white adipose tissue (WAT) and brown adipose tissue (BAT) depots. (**A**) Representative image of wild‐type (wt) periovarian WAT (periWAT). (**B**) Representative image of *Abcc5^‐/‐^* periWAT. (**C**) Representative image of wt interscapular BAT (iBAT). (**D**) Representative image of *Abcc5^‐/‐^* iBAT. (**E**) Total fat mass. Each data point represents mean ± SEM*.* Changes in total fat mass over time for *Abcc5^‐/‐^* vs. wt were analyzed by two‐way ANOVA with a Bonferroni post hoc test; ***P ≤ 0.001. Insert shows Western blot analysis of ABCC5 protein expression in adipose tissue. Lanes 1 and 2, periWAT from female *Abcc5^‐/‐^* mice and female wt mice, respectively; lanes 3 and 4, iBAT from female *Abcc5^‐/‐^* and female wt mice, respectively. (**F**) Mass of periWAT and (**G**) mass of iBAT; wt, *n* = 8; *Abcc5^‐/‐^*, *n* = 6 animals. (**H**) Lipid plasma levels for female *Abcc5^‐/‐^* mice (*n* = 14) and female wt mice (*n* = 15). Data shown as mean ± SEM; Welch's unequal variances *t* test; *P < 0.05; ***P < 0.001. [Color figure can be viewed at wileyonlinelibrary.com]

**Figure 3:**
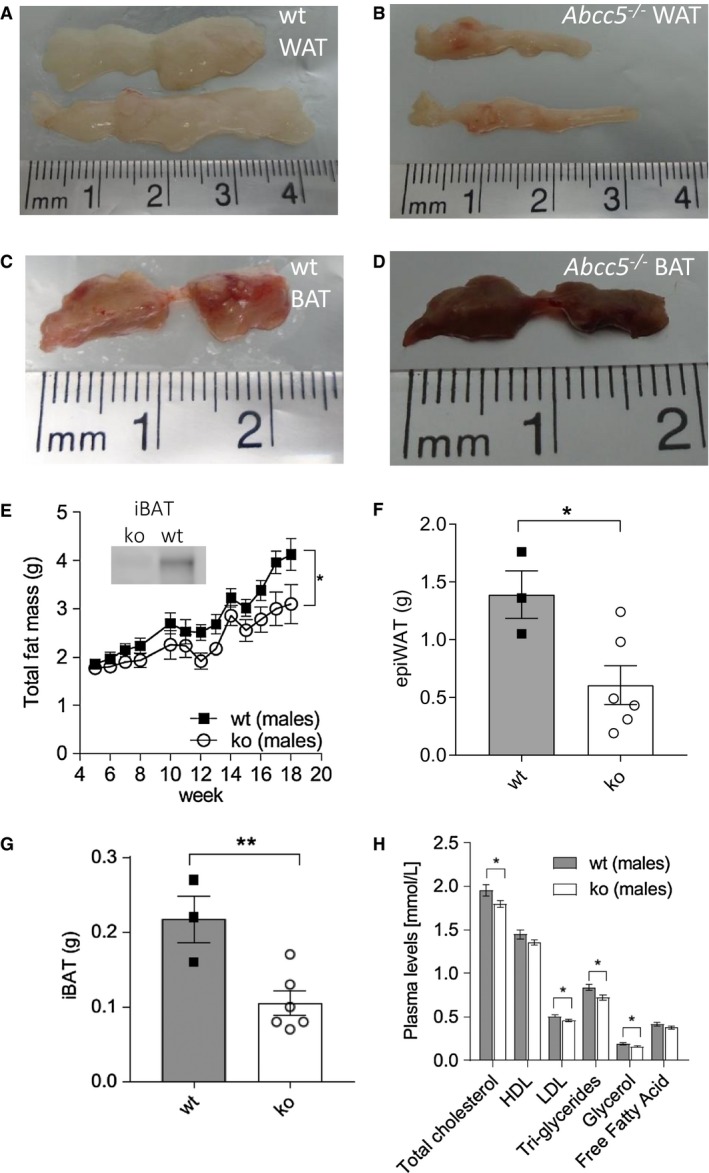
Male brown adipose tissue (BAT) and white adipose tissue (WAT) depots. (**A**) Representative image of wild‐type (wt) epididymal WAT (epiWAT). (**B**) Representative image of *Abcc5^‐/‐^* epiWAT. (**C**) Representative image of wt interscapular BAT (iBAT). (**D**) Representative image of *Abcc5^‐/‐^* iBAT. (**E**) Total fat mass. Each data point represents mean ± SEM*.* Changes in total fat mass over time for *Abcc5^‐/‐^* vs. wt were analyzed by two‐way ANOVA with a Bonferroni post hoc test; *P ≤ 0.05. Insert shows Western blot analysis of ABCC5 protein expression in adipose tissue. Lanes 1 and 2, iBAT for male *Abcc5^‐/‐^* and male wt mice, respectively. (**F**) Mass of epiWAT and (**G**) mass of iBAT; wt, *n* = 3, *Abcc5^‐/‐^*, *n* = 6 animals. (**H**) Lipid plasma levels for male *Abcc5^‐/‐^* mice (*n* = 15) and male wt mice (*n* = 15). Data shown as mean ± SEM; Welch’s unequal variances *t* test; *P < 0.05; **P < 0.01. [Color figure can be viewed at wileyonlinelibrary.com]

At 16 weeks, both female and male *Abcc5^‐/‐^* mice weighed ~10% less than wild‐type littermates, with a difference in body mass at 16 weeks of 2.5 g ± 0.8 g for *Abcc5^‐/‐^* females and 3.6 g ± 0.8 g for *Abcc5^‐/‐^* males (Figure 1A‐1B). Weekly EchoMRI results showed that *Abcc5^‐/‐^* mice had proportionally less fat (expressed as a fraction of total body mass, Figure 1C‐1D) and therefore more lean mass (expressed as a fraction of total body mass, Figure 1E‐1F), with changes more pronounced in female mice than males. Differences in weight were statistically significant from 5 weeks of age and for all weeks onward, as analyzed by a Student *t* test for wild‐type compared with *Abcc5^‐/‐^* mice for that week (Figure 1A‐1B). Changes in body composition over time shown in Figure 1 between wild‐type and *Abcc5^‐/‐^* mice were analyzed by two‐way ANOVA with a Bonferroni post hoc test, and significance values are indicated by asterisks shown at the end of the two curves. Notably, mice were of equal weight at weaning. Dual‐energy x‐ray absorptiometry scans performed at week 14 showed no difference in bone mineral density or bone mineral content, and x‐rays confirmed that there were no changes in femur length, showing that growth was not stunted in *Abcc5^‐/‐^* mice (data not shown). Both female and male *Abcc5^‐/‐^* mice had significantly lower masses of white adipose tissues, including periWAT depots in females and epiWAT depots in male mice (Figure 2A‐2B and 2F and Figure 3A‐3B and 3F). iBAT mass was significantly reduced in *Abcc5^‐/‐^* male mice (Figure 3C‐3D and 3G), and a similar trend was observed in iBAT of *Abcc5^‐/‐^* female mice (Figure 2C‐2D and 2G). Total fat mass in grams was decreased for both sexes (Figures 2E and 3E). Cholesterol and triglyceride plasma profiles showed decreased levels of total cholesterol, LDL, glycerol, and triglycerides in both female and male *Abcc5^‐/‐^* mice, which would suggest that *Abcc5^‐/‐^* mice did not have dyslipidemia (Figures 2H and 3H).

### *Abcc5^‐/‐^* mice are more active

**Figure 4:**
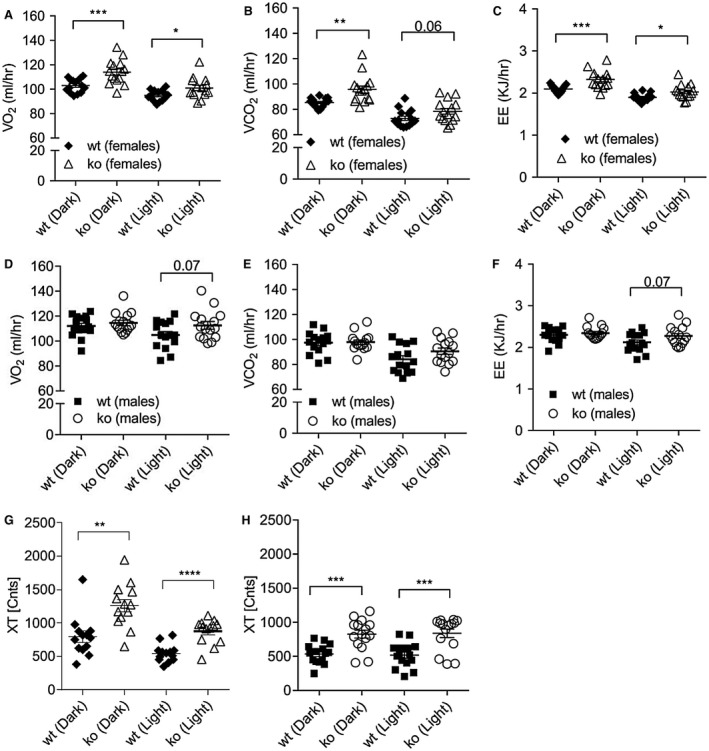
Indirect calorimetry and metabolic cage analysis of *Abcc5^‐/‐^* mice. (**A**) Female oxygen consumption (VO_2_). (**B**) Female carbon dioxide release (VCO_2_). (**C**) Female energy expenditure (EE). (**D**) Male oxygen consumption (VO_2_). (**E**) Male carbon dioxide release (VCO_2_). (**F**) Male EE. All data were ANCOVA adjusted for lean mass. Total activity (XT) for (**G**) females and (**H**) males. *Abcc5^‐/‐^* mice and wild‐type (wt) littermate controls (male:female:wt:*Abcc5^‐/‐^* = 15:11:15:15) were individually housed for 24 hours in PhenoMaster cages at 12 weeks of age. Data in panels G and H shown as the average XT activity per hour plotted separately for light‐dark cycle for each mouse. Error bars: mean ± SEM; unpaired two‐tailed Student *t* test; *P < 0.05; **P < 0.01; ***P < 0.001; ****P < 0.0001.

**Figure 5:**
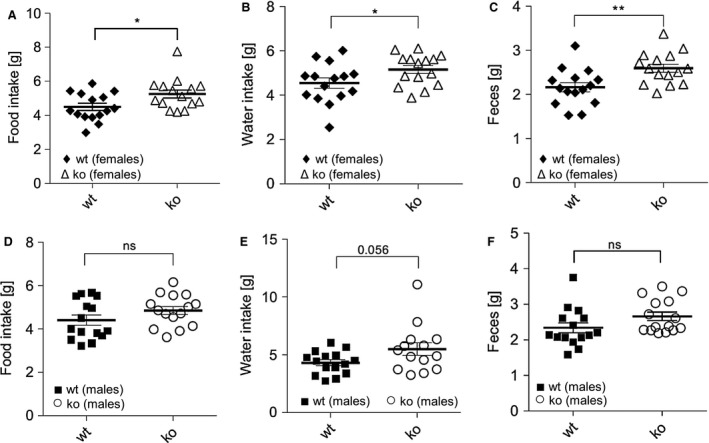
Food and water intake. (**A**) Food intake for females. (**B**) Water intake for females. (**C**) Fecal production for females. (**D**) Food intake for males. (**E**) Water intake for males. (**F**) Fecal production for males. Data shown as mean ± SEM, analyzed using an unpaired two‐tailed Student *t* test (male:female:wild‐type (wt):*Abcc5^‐/‐^* = 15:15:15:15); *P ≤ 0.05; **P ≤ 0.01.

Indirect calorimetry analysis found that female *Abcc5^‐/‐^* mice had increased VO_2_, CO_2_ release, and energy expenditure in both dark and light cycles, while male mice did not (Figure 4A‐4F). Both sexes of *Abcc5^‐/‐^* mice were more active in both dark and light cycles, showing increased total activity (the sum of ambulatory movement and fine movement) (Figure 4G‐4H). No changes in respiratory exchange ratio (RER) were observed for either sex (data not shown), which would indicate that the energy source used by *Abcc5^‐/‐^* mice was not switched from carbohydrate (standard chow RER = 0.9‐1.0) to fat (RER = 0.7). *Abcc5^‐/‐^* mice did not eat less, with female mice having slightly elevated food and water intake, while male *Abcc5^‐/‐^* mice showed no change (Figure 5). The decreased fat depots in *Abcc5^‐/‐^* mice can therefore not be explained by hypophagia in *Abcc5^‐/‐^* mice.

### *Abcc5^‐/‐^* mice are more insulin sensitive

**Figure 6:**
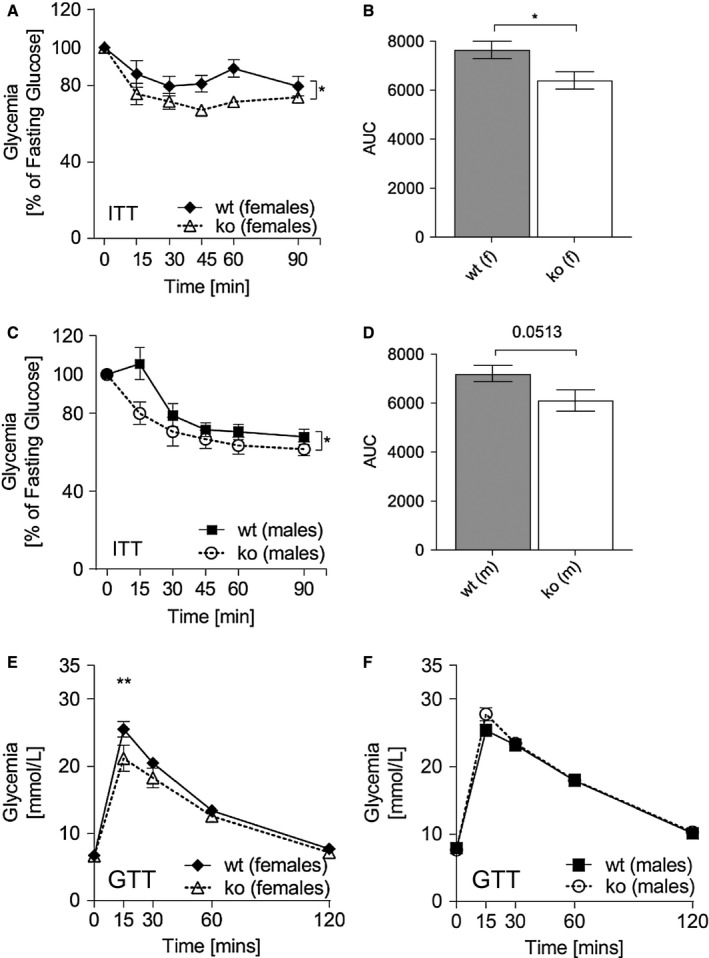
*Abcc5^‐/‐^* mice show increased insulin sensitivity. Insulin tolerance test (ITT) for (**A,B**) female and (**C,D**) male mice. ITT was performed on mice aged 14 weeks; male:female:wild‐type (wt):*Abcc5^‐/‐^* = 15:15:15:14. Data shown as mean ± SEM. Changes in plasma glucose in response to intraperitoneal insulin over time for *Abcc5^‐/‐^* vs. wt were analyzed by two‐way ANOVA with a Bonferroni post hoc test. Area under the curve (AUC) was analyzed by an unpaired two‐tailed Student *t* test; *P < 0.05. Intraperitoneal glucose tolerance tests (GTT) on 13‐week‐old (**E**) female and (**F**) male mice (male:female:wt:*Abcc5^‐/‐^* = 15:15:15:14). Data shown as mean ± SEM. Changes in plasma glucose in response to intraperitoneal glucose over time for *Abcc5^‐/‐^* vs. wt were analyzed by multiple comparison two‐way ANOVA with a Bonferroni post hoc test; **P < 0.01.

Female *Abcc5^‐/‐^* mice were able to lower plasma glucose more efficiently than wild‐type littermates in response to an intraperitoneal insulin bolus following a 4‐hour fast (Figure 6A‐6B), and the same trend was observed in male mice (Figure 6C‐6D). Changes in plasma glucose in response to intraperitoneal insulin over time for *Abcc5^‐/‐^* versus wild‐type mice were analyzed by two‐way ANOVA with a Bonferroni post hoc test (Figure 6A and 6C). The area under the curve were analyzed by an unpaired two‐tailed Student *t* test (Figure 6B and 6D). An intraperitoneal glucose tolerance test showed no phenotype‐dependent differences in response to glucose for either sex (Figure 6E‐6F). However, a small but significant increase in plasma glucose levels was observed at 15 minutes post glucose administration in female (Figure 6E) but not male *Abcc5^‐/‐^* mice (Figure 6F) when analyzed by multiple comparison two‐way ANOVA with a Bonferroni post hoc test. By contrast, no differences were observed in oral glucose tolerance in either sex (Supporting Information Figure S2). It is important to note that the increased insulin sensitivity observed in *Abcc5^‐/‐^* mice may therefore be secondary to the decreased adiposity of *Abcc5^‐/‐^* mice. By extension, it would appear that glucose‐stimulated insulin secretion in *Abcc5^‐/‐^* mice is not greatly affected, as plasma glucose levels following both intraperitoneal glucose tolerance test and oral glucose tolerance test showed little or no change when compared with wild‐type littermates. Future studies using a euglycemic hyperinsulinemic clamp with stable isotopic glucose and water tracers will be required to delineate the basis of increased insulin sensitivity in *Abcc5^‐/‐^* mice. Histological analysis of hematoxylin‐ and eosin‐stained gut, liver, and pancreas samples showed no discernible changes in tissue morphology of *Abcc5^‐/‐^* mice when compared with wild‐type littermates (data not shown).

### *Abcc5^‐/‐^* mice have raised plasma GLP‐1 levels

**Figure 7:**
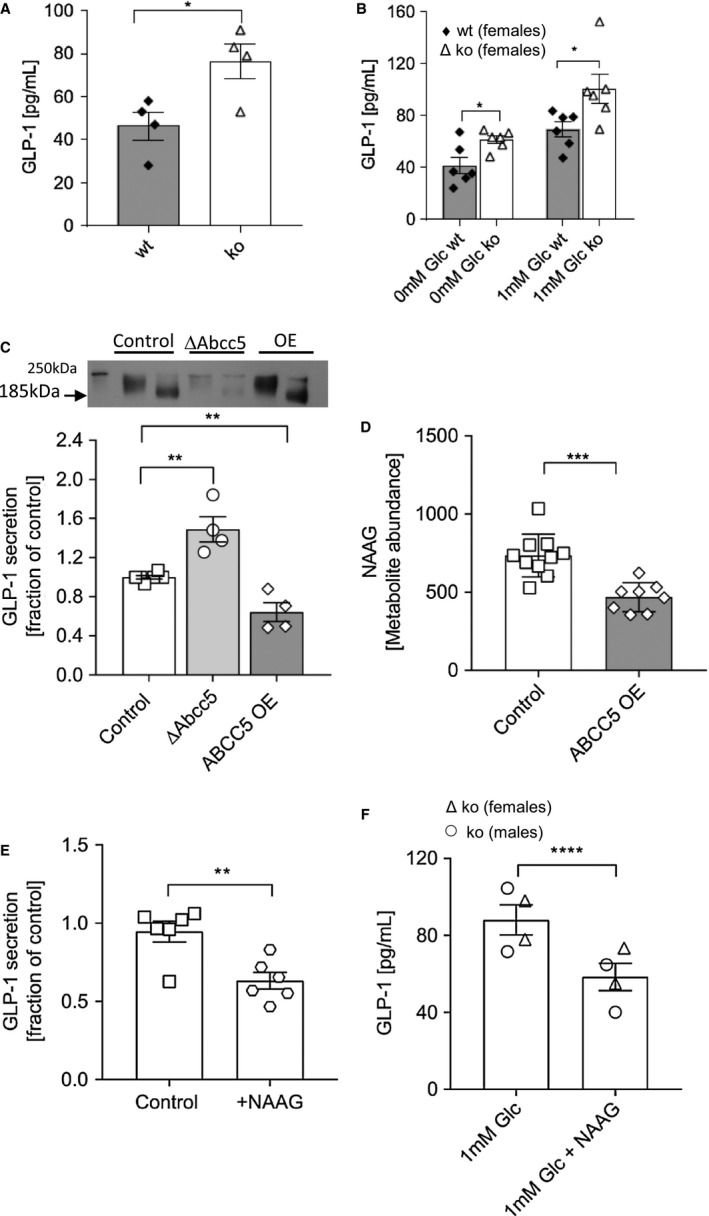
GLP‐1 exocytosis is dependent on ABCC5 protein levels. (**A**)* Abcc5^‐/‐^* mice (*n* = 4, female, open bar) had increased circulating levels of total plasma GLP‐1 when compared with wild‐type (wt) littermates (*n* = 4, female, filled bar) at 5 minutes following a fasted glucose challenge. (**B**) Active GLP‐1 secretion from *ex vivo* gut crypt primary cell cultures was increased in *Abcc5^‐/‐^* mice (*n* = 6, female, open bar) compared with wt littermates (*n* = 6, female, filled bar) in both the absence (0mM Glc) and presence of glucose (1mM Glc). (**C**) Total GLP‐1 secretion from GLUTag cells following a 2‐hour incubation in 5.6mM glucose (open squares, *n* = 6) showed increased GLP‐1 release from *Abcc5* siRNA treated GLUTag cells (open circles, *n* = 6), while overexpression (OE) of ABCC5 protein attenuated GLP‐1 release (open diamonds, *n* = 4). Data presented as a fraction of GLP‐1 secreted in 5.6mM (control) glucose. Data for panels A‐C shown as mean ± SEM and were analyzed by an unpaired Welch’s unequal variances *t* test; *P ≤ 0.05; **P ≤ 0.01. Insert is Western blot analysis of ABCC5 protein expression in wt GLUTag cells (lane 1, unheated sample; lane 2, heated sample), siRNA knockdown of *Abcc5* gene expression (lane 3, unheated sample; lane 4, heated sample), and recombinant OE of ABCC5 protein (lanes 5, unheated sample; lane 6, heated sample). ABCC5 typically migrates on an SDS‐PAGE gel at a molecular weight of 185 kDa in heated samples and at a higher molecular weight of about 200 kDa in unheated samples. (**D**)* ABCC5* OE (open diamonds) decreased levels of NAAG in GLUTag cells when compared with control (open squares); control, *n* = 10; *ABCC5* OE, *n* = 8. (**E**) Exogenous NAAG inhibited secretion of GLP‐1 from GLUTag cells (open hexagons, *n* = 6) when compared with control (open squares, *n* = 6) in the presence of 1mM glucose. Data for panels D and E shown as mean ± SEM and were analyzed by an unpaired two‐tailed Student *t* test; **P ≤ 0.01; ***P ≤ 0.001. (**F**) Exogenous NAAG inhibited secretion of GLP‐1 from *ex vivo* gut crypt primary small intestine cultures of *Abcc5^‐/‐^* mice (*n* = 4); ****P ≤ 0.0001; unpaired Welch’s unequal variances *t* test. Error bars: mean ± SEM.

Circulating plasma levels of total GLP‐1 measured 5 minutes following an oral glucose gavage were increased by 60% in *Abcc5^‐/‐^* mice compared with wild‐type littermates (Figure 7A). We were unable to measure baseline GLP‐1 levels from plasma from mice fasted overnight, and only postprandial GLP‐1 levels could be detected. To determine the source of increased GLP‐1 detected in plasma, *ex vivo* gut crypt primary cell cultures were grown from *Abcc5^‐/‐^* mouse small intestine and analyzed for GLP‐1 secretion at rest and in the presence of glucose. Recapitulating the plasma results, *Abcc5^‐/‐^* mouse gut primary cells secreted more active GLP‐1 in both the presence and absence of glucose when compared with wild‐type littermates (Figure 7B).

### *Abcc5* is expressed in mouse enteroendocrine cells

Transcriptional profiling of the enteroendocrine cells of mouse duodenum, ileum, and colon showed increased gene expression of *Abcc5* in the enteroendocrine cells of the duodenum and in preproglucagon‐expressing L‐cells of the ileum when compared with nonendocrine cells (Table 1). GLUTag cells, a model L‐cell line, expressed levels of *Abcc5* mRNA similar to that observed in the enteroendocrine cells of the duodenum and ileum (Table 1). Interestingly, low‐resolution fluorescent microscopy images indicated that ABCC5 was not expressed in the apical or basolateral membranes of enteroendocrine cells, and protein expression was shown to be intracellular in both GLUTag cells (Supporting Information Figure S3A) and gut L‐cells from GLU‐Venus mice (Supporting Information Figure S3B).

**Table 1:** Expression of *Abcc5* in mouse EEC

	FPKM (gut endocrine)	FPKM (nonendocrine)	log2FC positive/negative (*P *adjusted)
**Duodenum_EEC**	9.739	1.903	2.050 (0.001)
**Ileum_EEC**	8.851	3.389	0.884 (0.414)
**Colon_EEC**	3.676	2.932	0.809 (0.640)
**Duodenum_L‐cells**	7.939	3.350	1.525 (0.228)
**Ileum_L‐cells**	12.353	2.039	1.928 (0.019)
**Colon_L‐cells**	4.456	4.800	1.357 (0.116)
**GLUTag cells**	6.491		

Transcriptional profiling of *Abcc5* expression in different regions of mouse gastrointestinal tract and GLUTag cells, expressed in FPKM. Fold enrichment (expressed as log2) and confidence of the enrichment (*P *adjusted) determined on normalized data using a differential expression analysis (DESeq) model.

EEC, enteroendocrine cells; FPKM, fragments per kilobase per million read.

### GLP‐1 exocytosis from gut enteroendocrine cells is inversely dependent on ABCC5 protein expression levels

In order to test a direct link between ABCC5 protein expression and GLP‐1 secretion, we used a well‐characterized model L‐cell line, GLUTag cells, which secrete GLP‐1 in response to stimulation by nutrients 23, 24. siRNA knockdown of *Abcc5* gene expression in GLUTag cells resulted in a ~60% increase in active GLP‐1 release (Figure 7C), while recombinant overexpression of *ABCC5* attenuated GLP‐1 release below the exocytosis levels observed in the untreated control. ABCC5 protein expression levels in GLUTag cells used for GLP‐1 secretion assays were confirmed by Western blot (Figure 7D). Western blot analysis of wild‐type GLUTag cells showed robust ABCC5 protein expression (Figure 7C, lanes 1 and 2), while siRNA knockdown of *Abcc5* gene expression in GLUTag cells reduced ABCC5 protein expression levels substantially (Figure 7C, lanes 3 and 4); recombinant overexpression of ABCC5 protein was also confirmed (Figure 7C, lanes 5 and 6).

Using metabolomics, we identified a known ABCC5 substrate, NAAG, as an abundant glutamate metabolite in GLUTag cells. In order to confirm that the cellular levels of NAAG were also inversely related to *ABCC5* expression in GLUTag cells, similar to that previously observed in human embryonic kidney (HEK) cells, *ABCC5* was overexpressed in GLUTag cells and the levels of NAAG analyzed by comparative metabolomics 18. The intracellular levels of NAAG were decreased in the *ABCC5*‐overexpressing GLUTag cells when compared with sham‐transfected GLUTag cells (Figure 7D). To investigate a potential role of this inhibitory neuropeptide in ABCC5‐mediated regulation of gut hormone release, the effects of exogenous NAAG on GLP‐1 levels were measured. The exogenous addition of 2mM NAAG to both GLUTag cells and *ex vivo* gut crypt primary cell cultures, generated from *Abcc5^‐/‐^* mice, inhibited the release of GLP‐1 (Figure 7E‐7F). Taken together, this data would suggest that ABCC5 activity modulates GLP‐1 release from gut endocrine cells through a NAAG‐dependent mechanism.

## Discussion

The most prominent metabolic phenotype of *Abcc5^‐/‐^* mice is a decrease in total levels of fat mass. Transcriptional profiling of human subcutaneous adipose tissue by Direk et al. 8 showed high levels of *ABCC5* gene expression and demonstrated that elevated expression of *ABCC5* in subcutaneous adipose tissue confers an increased risk for developing T2D with age in populations of disparate ancestry. The prevalence of T2D was reported to be three times higher in subjects with high *ABCC5* expression in subcutaneous adipose tissue compared with those with low expression, and overexpression was most strongly associated with increased visceral white adipose tissue accumulation and reduced peripheral insulin sensitivity in nondiabetic individuals. Interestingly, the human overexpression phenotype is the opposite of our global *Abcc5^‐/‐^* mice, in which both sexes displayed decreased fat mass and increased insulin sensitivity. Adipose tissue is regulated by multiple endocrine and neurocrine inputs, and *Abcc5^‐/‐^* mice showed decreases in both white adipose tissue (periWAT in females and epiWAT in males) and iBAT in the absence of hypophagia (i.e., *Abcc5^‐/‐^* mice did not eat less). *Abcc5^‐/‐^* mice were not burning fat in response to adaptive thermogenesis, as there was no difference between the RER of *Abcc5^‐/‐^* and wild‐type littermates and no browning was observed in the white adipose deposits of *Abcc5^‐/‐^* mice (data not shown). Both male and female mice were more active overall, but only in the females was increased activity coupled to increased energy expenditure as reflected in increased VO_2_. Increased energy expenditure could therefore contribute to the decreased fat deposits observed in female mice. On the other hand, male mice appeared to have increased activity and decreased fat mass in the absence of changes in energy consumption (VO_2_), food intake, or adaptive thermogenesis. It was previously shown that more‐active mice did not expend more energy under standard laboratory conditions (i.e., below thermoneutrality) 25. The decreased fat deposits in male *Abcc5^‐/‐^* mice would therefore suggest an additional role for ABCC5 in adipocyte physiology.

In addition to decreased fat mass, raised circulating plasma levels of GLP‐1 were also observed in *Abcc5^‐/‐^* mice. Raised plasma GLP‐1 appears to be the result of increased GLP‐1 release from gut endocrine cells, as *ex vivo* primary gut crypt cultures from *Abcc5^‐/‐^* mice also showed increased GLP‐1 release, both at rest and in response to stimulation by glucose. Furthermore, an inverse relationship between ABCC5 protein expression and GLP‐1 release was also confirmed in a model L‐cell line, GLUTag cells, in which recombinant overexpression of ABCC5 suppressed GLP‐1 secretion. ABCC5 has previously been identified as an amino acid conjugate transporter and it exports both *N*‐lactoyl amino acids and glutamate‐aspartate conjugates from stably transfected HEK cells 18, 26. Jansen et al. 18 demonstrated the accumulation of eight glutamate conjugates in the tissues of *Abcc5^‐/‐^* mice using untargeted metabolomics, some of which are inhibitory neurotransmitters. Notably, the most abundant metabolite, NAAG, is a glutamate neurotransmission antagonist, and NAAG action downregulates excitatory glutamatergic neurons 27, 28. Glutamate also stimulates GLP‐1 release from gut endocrine cells; it has been previously shown that glutamate and GLP‐1 are loaded into the same vesicles in intestinal L‐cells, and exogenous glutamate administration stimulates GLP‐1 release from GLUTag cells 24, 29.

Here, we were able to duplicate the HEK cell work done by Jansen et al. 18 in GLUTag cells and demonstrated that ABCC5 also acts as a NAAG exporter in enteroendocrine cells. Furthermore, exogenous addition of NAAG inhibits GLP‐1 release from both GLUTag cells and primary gut crypts. Intriguingly, as native ABCC5 protein expression in both GLUTag cells and L‐cells of the ileum appears to be intracellular, and ABCC5 is a NAAG exporter, a possible explanation could be that ABCC5 is involved in loading this neuropeptide into synaptic‐like vesicles of enteroendocrine cells, which are released upon exocytosis. The transport of glutamate and neuropeptides into both distinct and overlapping vesicle pools is well described in neurons, but little is known about how NAAG is loaded into the synaptic vesicles in the brain 30. Therefore, if ABCC5 transporter activity is indeed involved in the export of inhibitory neurotransmitters such as NAAG, loss of this transporter could lead to a general increase in glutamatergic activation, such as is observed in the increase in total activity of both male and female mice as well as increased GLP‐1 release from enteroendocrine cells. However, it has been shown that when recombinantly overexpressed *in vitro*, in addition to glutamate metabolites, ABCC5 may also transport organic anions (such as 6‐mercaptopurine and thioguanine), pyrimidine‐based antivirals such as 2’‐3’‐dideoxynucleotides, folates, various cyclic nucleotides, and N‐lactoyl amino acids. We therefore cannot exclude the possibility that any of these other substrates may also be involved in the regulation of GLP‐1 secretion from L‐cells 26, 31, 32, 33, 34, 35, 36.

In the brain, ABCC5 protein expression was localized to astrocytes of the subcortical white matter as well as to pyramidal neurons 37. Interestingly, a recent report attributed the weight loss observed following the administration of the GLP‐1R agonist liraglutide to increased glutamatergic signaling in the brain 38. GLP‐1R activation in *Abcc5^‐/‐^* mice could therefore be upregulated through both increased circulating levels of GLP‐1 and increased glutamatergic signaling caused by a loss of NAAG inhibition.

In summary, *Abcc5^‐/‐^* mice have a surprisingly complex metabolic phenotype, are lean, have increased circulating plasma levels of GLP‐1, and are more insulin sensitive. *Abcc5^‐/‐^* mice are the opposite of the observed human overexpression phenotype, which is associated with increased visceral fat, insulin resistance, and a susceptibility to T2D with age. This study confirmed an important role for ABCC5 in adipocyte physiology in mammals. Future studies using inducible tissue‐specific *Abcc5^‐/‐^* mice housed at thermoneutrality are now needed to dissect the metabolic implications of ABCC5 protein loss in the gut, the brain, the pancreas, and adipose tissue.
