# Supplementary material for: Abcc5 Knockout Mice Have Lower Fat Mass and Increased Levels of Circulating GLP‐1
Source: Obesity (Silver Spring). 2019 Jul 24;27(8):1292–304. doi: 10.1002/oby.22521 (PMC6658130; doi:10.1002/oby.22521)
Supplement: Supplementary file 1 [file OBY-27-1292-s001.pdf]

Supporting Information

Methods

Adipose tissue harvest and Western Blots

Total protein from WAT and BAT tissues was extracted by mechanical homogenisation using a pestle and mortar. Each tissue sample was homogenised with 1mL ice-cold lysis buffer containing 50mM Tris-HCl (pH 7.5) (Sigma Aldrich), 1mM EGTA (pH 8) (Alfa Aesar), 1mM EDTA (pH 8) (Alfa Aesa)], 1% Triton X-100 (Sigma Aldrich), 1mM Sodium Orthovanadate (Sigma Aldrich), 50mM Sodium Fluoride (Sigma Aldrich), 5mM Sodium Pyrophosphate (Sigma Aldrich), 0.27M Sucrose (Sigma Aldrich), 1mM PMSF (Thermo Fisher Scientific) and Complete protease inhibitor cocktail (1 tablet/50 mL) (Roche Diagnostics)]. Homogenized samples were centrifuged at 13,000rpm for 25min at +4°C. Supernatants were collected (excluding the pellet and top layer of fat) and stored at -80°C until quantification and subsequent SDS-PAGE and Western blot. Antibodies were goat polyclonal anti-ABCC5, sc-5781, Santa Cruz Biotechnology, 1:200; secondary goat anti-Rabbit IgG (H/L):HRP, STAR124P, AbD Serotec, 1:10,000).

Isolation and culture of gut primary cells from *Abcc5*$^{-/-}$ mice

Briefly, for primary gut cell cultures the middle part of murine small intestine (10cm lower down from the duodenum) was dissected and cut open longitudinally, washed thoroughly with cold PBS (Ca^{2+}/Mg^{2+}), followed by fine dicing of the tissue using a scalpel. The tissue fragments underwent several collagenase digests at 37°C (C9407-10MG, Sigma), where the top crypt-enriched fraction was collected into 15mL tubes and centrifuged at 300xg for 5min. Pelleted crypts were resuspended in the growth media: DMEM (D6546-500ML, Sigma) supplemented with 100U/mL Penicillin and 100ug/mL Streptomycin (15140122, Gibco), 10% Foetal Bovine Serum (F7524-500ML, Sigma), 2mM Glutamine, 10uM ROCK inhibitor (Y27632), and passed through a 100uM strainer. Combined fractions from 4-5 collagenase digests were pooled together and plated onto Matrigel coated 24-well plates in the growth media mentioned above and grown at 37°C, 5%CO_2 for at least 16-18 hours prior secretion experiments.

Metabolomics

Metabolite extraction from cells: Metabolites were extracted from approximately $5x10^6$ cells (grown in cell culture dishes) by addition of 500µL of ice cold 80% aqueous methanol. The supernatants were combined and filtered using a 3kDa ultrafilter (Millipore), dried in a SpeedVac and subsequently stored at -80°C. On the day of analysis, the dried extracts were re-constituted in 60µL of ice cold 80% aqueous methanol. A quality control (QC) sample was made by combing 5µL of each sample. This was injected at the start of the sequence and subsequently every 10 samples throughout the LC-MS/MS analyses.

Metabolomics: LC-MS/MS analysis: Each sample was analysed using two separate LC-MS/MS methods utilising two separate chromatographic systems (Thermo Scientific ICS-5000+ ion chromatography system and a Thermo Ultimate 3000, Thermo Scientific, San Jose, CA). Each was coupled directly to the same Q-Exactive HF Hybrid Quadrupole-Orbitrap mass spectrometer with a HESI II electrospray ionisation source (Thermo Scientific, San Jose, CA).

Ion exchange chromatography was performed using a ICS-5000+ HPLC system incorporating an electrolytic anion generator (KOH) which was programmed to produce a OH– gradient over 37min. An inline electrolytic suppressor removed OH– ions and cations from the post-column eluent stream prior to MS analysis (Thermo Scientific Dionex AERS 500). A 10µL partial loop injection was used for all analyses and the chromatographic separation was performed using a Thermo Scientific Dionex IonPac AS11-HC 2 × 250 mm, 4µm particle size column with a Dionex Ionpac AG11-HC 4 µm 2x50 guard column inline. The IC flow rate was 0.250mL/min. The total run time was 37min and the hydroxide ion gradient comprised as follows: 0min, 0mM; 1min, 0mM; 15min, 60mM; 25min, 100mM; 30min, 100mM; 30.1min, 0mM; 37min, 0mM. Analysis was performed in negative ion mode using a scan-range from m/z 60-900 and resolution set to 70,000. The tune file source parameters were set as follows: Sheath gas flow 60mL/min; Aux gas flow 20mL/min; Spray voltage 3.6v; Capillary temperature 320°C; S-lens RF value 70; Heater temperature 350°C. AGC target was set to 1e6v ions and the Max IT value was 250ms. The column temperature was kept at 30°C throughout the experiment. Full scan data were acquired in continuum mode.

C18 reversed-phase analysis was performed using a Thermo Utimate 3000 UHPLC system with a gradient elution program coupled directly to a Q-Exactive HF Hybrid Quadrupole-Orbitrap mass spectrometer. A 5 µL partial loop injection was used for all analyses with pre and post injection wash program. A Waters CORTECS UPLC T3 1.6µm (2.1x100mm) column was used with a flow rate of 0.4mL/min. The total run time was 18min. Mobile phase A comprised milli-Q water with 0.1% formic acid and mobile phase B was 100% methanol with 0.1% formic acid. The gradient elution program was as follows: 0mins, 5%B; 4min, 50%B; 12min, 99%B; 15min, 99%B; 15.1min, 5%B; 18min, 5%B. The column temperature was kept at 40°C throughout the experiment. Mass spectrometry analysis was performed in positive and negative ion mode separately using a scan-range from m/z 60-900 and resolution set to 70,000. The tune file source parameters were set as follows: Sheath gas flow 60 mL/min; Aux gas flow 20mL/min; Spray voltage 3.6v; Capillary temperature 320°C; S-lens RF value 70; Heater temperature 350°C. Full MS setting were AGC target 5e6 ions and the Max IT value was 120ms. Full scan data were acquired in continuum mode. A data directed tandem mass spectrometry method was utilised (ddMS2) with no inclusion list. The orbitrap detector and HCD setting for ddMS2 were as follows: Microscans 2, resolution 17,500, AGC target 5e4 ions, maximum IT 80ms, loop count 10 and NCE 35.

Data processing: Raw data files were processed using ProgenesisQI (Waters, Elstree, UK). This involved alignment of retention times, peak picking by identification of the presence of natural abundance isotope peaks, characterising multiple adducts forms and identification of metabolites using our in house database of authentic standards. Retention times, accurate mass values, relative isotope abundances and fragmentation patterns were compared between authentic standards and the samples measured. Identifications were accepted only when the following criteria were met: <5ppm differences between measured and theoretical mass (based on chemical formula), <30s differences between authentic standard and analyte retention times, isotope peak abundance measurements for analytes were >90% matched to the theoretical value generated from the chemical formula. Where measured, fragmentation patterns were matched to least the base peak and two additional peak matches in the MS/MS spectrum to within 12ppm. The top 10 data directed fragmentation method was not always able to provide fragment ions for all ions measured in the MS 1 spectrum.

Data analysis: Principal Component Analysis (PCA) was performed using the capability for this output in Progenesis QI. Fold change, %CV and p-values were generated automatically in progenesis QI and verified manually using a normalised abundance output and Excel. Heat maps were generated manually using the verified fold-change output.

Immunofluorescence imaging of GLUTag cells and GLUVenus mouse ileum

GLUTag cells were grown on glass cover slips coated with 2% Matrigel until 80% confluent, fixed in 10% neutral buffered formalin (NBF) for 30min and processed for imaging according to standard protocols. GLUVenus mouse ileum were harvested in ice cold PBS, fixed in 10% NBF for 2h and processed for wax embedding according to standard protocols. ABCC5 was visualized by primary antibody goat polyclonal anti-Abcc5 (SC5781, Santa Cruz biotechnology) at 1:50 dilution and GLP-1 by primary antibody rabbit polyclonal anti-GLP-1 (LS-B9319, LSBio) at 1:50 dilution. Secondary antibodies were donkey anti-goat IgG (H+L) Alexa Fluor® 488 (Life Technologies A11055) and donkey anti-rabbit IgG H&L Alexa Fluor® 568 at 1:500 (ab175692, Abcam).

Figure S1

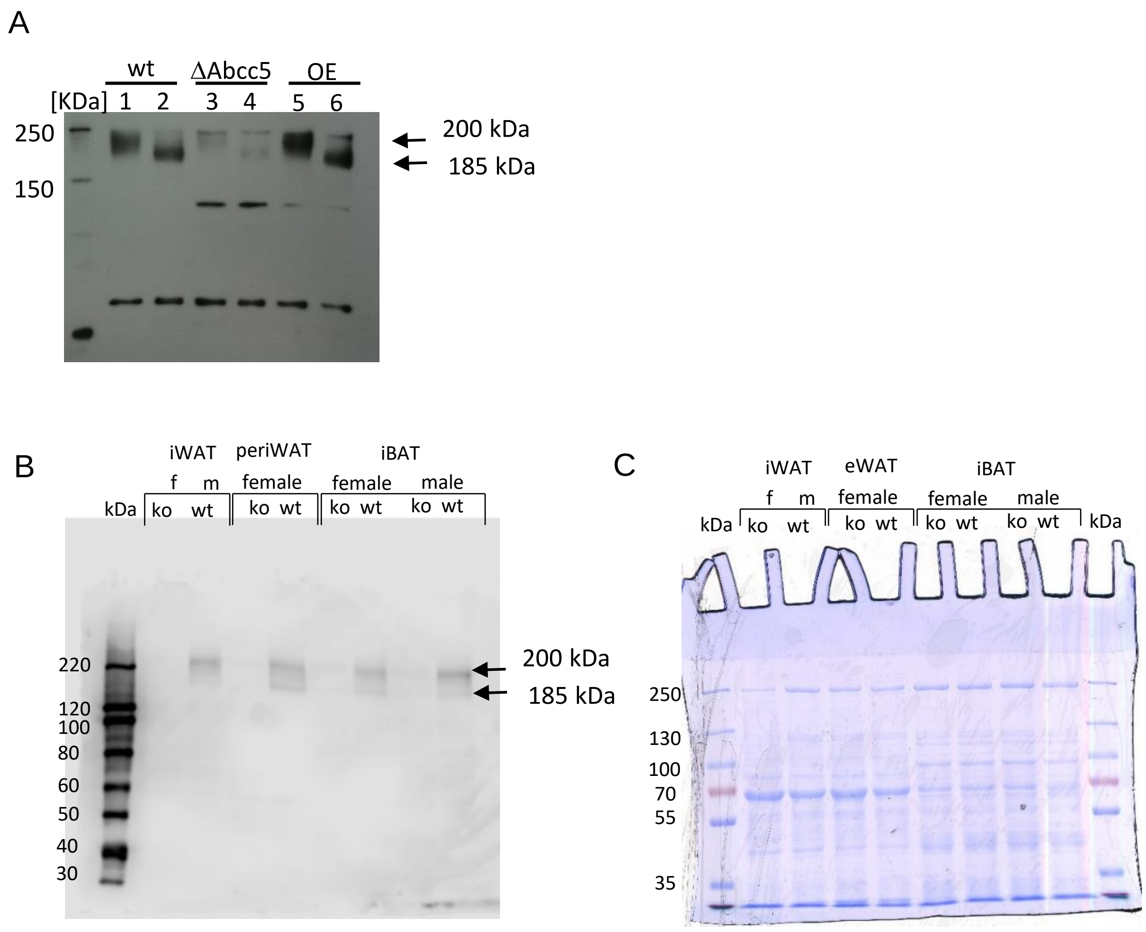

Figure S1. Full Western blot of ABCC5 protein expression. (A) Western Blot analysis of wt GLUTag cells shows expression of ABCC5 at an expected molecular weight of 185kDa (lane 1, unheated sample; lane 2, heated sample), siRNA knockdown of *Abcc5* gene expression (lanes 3, unheated sample; lane 4, heated sample) and recombinant overexpression of ABCC5 protein (lanes 5, unheated sample; lane 6, heated sample). ABCC5 typically migrates on a SDS-PAGE gel at a molecular weight of 185 kDa in heated samples and at a higher molecular weight of about 200 kDa in unheated samples. ***(B)*** Western Blot of ABCC5 protein expression in white adipose tissue (WAT) and brown adipose tissue (BAT) of *Abcc5*$^{-/-}$ (ko) mice and littermate wild type (wt) controls. Lane 1 (kDa), molecular weight marker (MagicMark XP Western Protein standard, Thermo Fisher Scientific); lanes 2 and 3, inguinal WAT (iWAT) from male *Abcc5*$^{-/-}$ and female wt mice respectively; lanes 4 and 5, periovarian WAT (periWAT) from female *Abcc5*$^{-/-}$ and female wt mice respectively; lanes 6 and 7, intrascapular BAT (iBAT) from female *Abcc5*$^{-/-}$ and female wt mice respectively; lanes 8 and 9, iBAT for male *Abcc5*$^{-/-}$ and male wt mice. **(B)** A corresponding gel loaded as in (A) stained with Coomassie blue confirms equal loading and sample integrity.

Figure S2

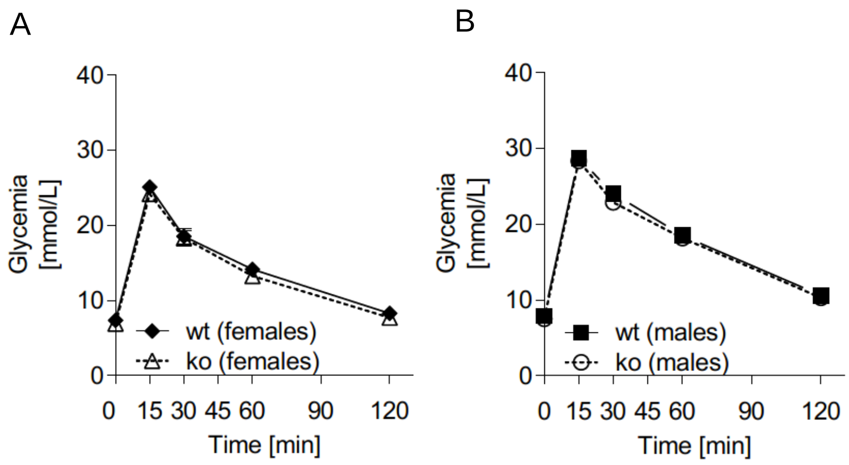

SI Figure 2. Oral glucose tolerance tests (OGTT). ***(A)*** female and ***(B)*** male *Abcc5*$^{-/-}$ (ko) mice and wild-type (wt) littermate controls. OGTT were performed on 12 week old mice (male:female:wt:*Abcc5*$^{-/-}$=15:15:15:14). Data shown as mean±SEM.

Figure S3

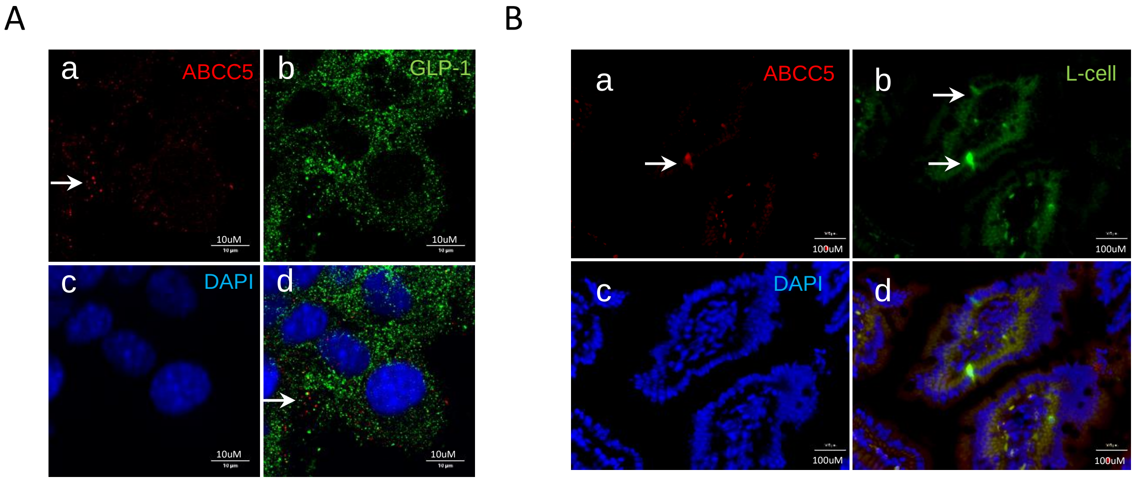

Figure S3. ABCC5 protein expression do not localize to the extracellular membrane. ***(A)*** ABCC5 protein expression (red) in GLUTag cells shows punctate, intracellular localization and did not co-localize with GLP-1 containing vesicles (green). Nuclei are visualized by DAPI (blue). ***(B)*** ABCC5 protein expression (red) is observed in GLP-1 expressing enteroendocrine cells (green) in GLUVenus mouse ileum. Nuclei were visualized by DAPI (blue).
